# Supplementary material for: Causal Relationship between Aging and Anorexia Nervosa: A White-Matter-Microstructure-Mediated Mendelian Randomization Analysis
Source: Biomedicines. 2024 Aug 16;12(8):1874. doi: 10.3390/biomedicines12081874 (PMC11351342; doi:10.3390/biomedicines12081874)
Supplement: Supplementary file 1 [file biomedicines-12-01874-s001.zip › Supplementary Figure/Figure S4/Singe SNP plot of MR analysis for TL to ubm-b-1932.pdf]

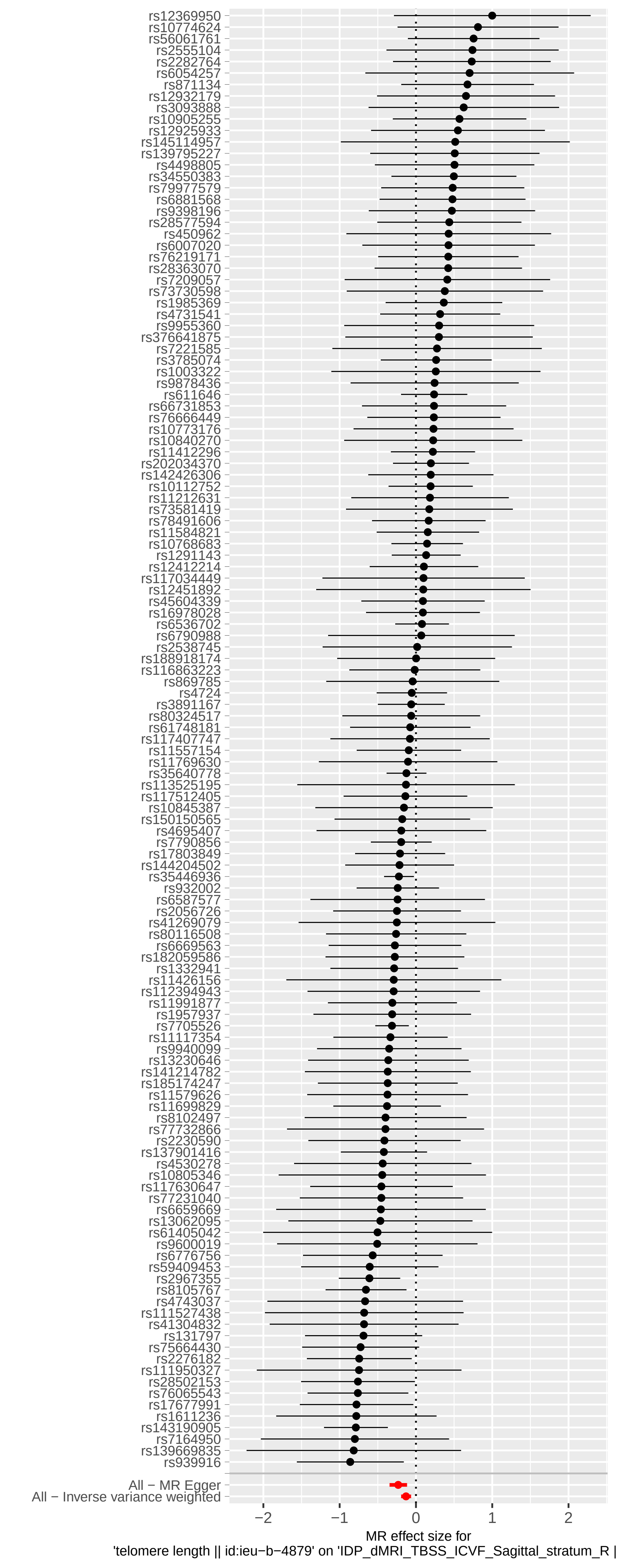
rs12369950
rs10774624
rs56061761
rs2555104
rs2282764
rs6054257
rs871134
rs12932179
rs3093888
rs10905255
rs12925933
rs145114957
rs139795227
rs4498805
rs34550383
rs79977579
rs6881568
rs9398196
rs28577594
rs450962
rs6007020
rs76219171
rs28363070
rs7209057
rs73730598
rs1985369
rs4731541
rs9955360
rs376641875
rs7221585
rs3785074
rs1003322
rs9878436
rs611646
rs66731853
rs76666449
rs10773176
rs10840270
rs11412296
rs202034370
rs142426306
rs10112752
rs11212631
rs73581419
rs78491606
rs11584821
rs10768683
rs1291143
rs12412214
rs117034449
rs12451892
rs45604339
rs16978028
rs6536702
rs6790988
rs2538745
rs188918174
rs116863223
rs869785
rs4724
rs3891167
rs80324517
rs61748181
rs117407747
rs11557154
rs11769630
rs35640778
rs113525195
rs117512405
rs10845387
rs150150565
rs4695407
rs7790856
rs17803849
rs144204502
rs35446936
rs932002
rs6587577
rs2056726
rs41269079
rs80116508
rs6669563
rs182059586
rs1332941
rs11426156
rs112394943
rs11991877
rs1957937
rs7705526
rs11117354
rs9940099
rs13230646
rs141214782
rs185174247
rs11579626
rs11699829
rs8102497
rs77732866
rs2230590
rs137901416
rs4530278
rs10805346
rs117630647
rs77231040
rs6659669
rs13062095
rs61405042
rs9600019
rs6776756
rs59409453
rs2967355
rs8105767
rs4743037
rs111527438
rs41304832
rs131797
rs75664430
rs2276182
rs111950327
rs28502153
rs76065543
rs17677991
rs1611236
rs143190905
rs7164950
rs139669835
rs939916
All − MR Egger
All − Inverse variance weighted
−2
−1
0
1
2
MR effect size for
'telomere length || id:ieu−b−4879' on 'IDP_dMRI_TBSS_ICVF_Sagittal_stratum_R |
